# Rapid Detection of Heterogeneous Vancomycin-Intermediate *Staphylococcus aureus* Based on Matrix-Assisted Laser Desorption Ionization Time-of-Flight: Using a Machine Learning Approach and Unbiased Validation

**DOI:** 10.3389/fmicb.2018.02393

**Published:** 2018-10-11

**Authors:** Hsin-Yao Wang, Chun-Hsien Chen, Tzong-Yi Lee, Jorng-Tzong Horng, Tsui-Ping Liu, Yi-Ju Tseng, Jang-Jih Lu

**Affiliations:** ^1^Department of Laboratory Medicine, Chang Gung Memorial Hospital at Linkou, Taoyuan, Taiwan; ^2^Ph.D. Program in Biomedical Engineering, Chang Gung University, Taoyuan, Taiwan; ^3^Department of Information Management, Chang Gung University, Taoyuan, Taiwan; ^4^Department of Computer Science and Engineering, Yuan Ze University, Taoyuan, Taiwan; ^5^Innovation Center for Big Data and Digital Convergence, Yuan Ze University, Taoyuan, Taiwan; ^6^Warshel Institute for Computational Biology, Chinese University of Hong Kong, Shenzhen, China; ^7^School of Science and Engineering, Chinese University of Hong Kong, Shenzhen, China; ^8^Department of Bioinformatics and Medical Engineering, Asia University, Taichung, Taiwan; ^9^Department of Computer Science and Information Engineering, National Central University, Taoyuan, Taiwan; ^10^School of Medicine, Chang Gung University, Taoyuan, Taiwan; ^11^Department of Medical Biotechnology and Laboratory Science, Chang Gung University, Taoyuan, Taiwan

**Keywords:** heterogeneous vancomycin-intermediate *Staphylococcus aureus*, matrix-assisted laser desorption ionization (MALDI) mass spectrometry, vancomycin intermediate *S. aureus* (VISA), machine learning, rapid detection

## Abstract

Heterogeneous vancomycin-intermediate *Staphylococcus aureus* (hVISA) is an emerging superbug with implicit drug resistance to vancomycin. Detecting hVISA can guide the correct administration of antibiotics. However, hVISA cannot be detected in most clinical microbiology laboratories because the required diagnostic tools are either expensive, time consuming, or labor intensive. By contrast, matrix-assisted laser desorption ionization time-of-flight (MALDI-TOF) is a cost-effective and rapid tool that has potential for providing antibiotics resistance information. To analyze complex MALDI-TOF mass spectra, machine learning (ML) algorithms can be used to generate robust hVISA detection models. In this study, MALDI-TOF mass spectra were obtained from 35 hVISA/vancomycin-intermediate *S. aureus* (VISA) and 90 vancomycin-susceptible *S. aureus* isolates. The vancomycin susceptibility of the isolates was determined using an Etest and modified population analysis profile–area under the curve. ML algorithms, namely a decision tree, k-nearest neighbors, random forest, and a support vector machine (SVM), were trained and validated using nested cross-validation to provide unbiased validation results. The area under the curve of the models ranged from 0.67 to 0.79, and the SVM-derived model outperformed those of the other algorithms. The peaks at m/z 1132, 2895, 3176, and 6591 were noted as informative peaks for detecting hVISA/VISA. We demonstrated that hVISA/VISA could be detected by analyzing MALDI-TOF mass spectra using ML. Moreover, the results are particularly robust due to a strict validation method. The ML models in this study can provide rapid and accurate reports regarding hVISA/VISA and thus guide the correct administration of antibiotics in treatment of *S. aureus* infection.

## Introduction

Methicillin-resistant *Staphylococcus aureus* (MRSA) infection remains an intractable clinical problem (Liu et al., 2011). Although vancomycin was formerly the drug of choice against MRSA, the unprecedented increase in the number and spread of organisms with reduced susceptibility to this drug, including two major phenotypes—vancomycin-intermediate *S. aureus* (VISA) and heterogeneous VISA (hVISA)—has brought this conventional treatment into question (Zhang et al., 2015). The prevalence of hVISA and VISA was reported in a systematic review to have increased worldwide from 4.68 and 2.05% (2006) to 7.01 and 7.93% (2014), respectively (Zhang et al., 2015). In Taiwan, the prevalence of hVISA also increased from 0.7% (2003) to 10.0% (2013) and that of VISA from 0.2% (2003) to 2.7% (2013) (Huang et al., 2016). Despite adequate doses of vancomycin, patients with severe hVISA or VISA infection persistently suffer from bacteremia (Howden et al., 2010). In particular, hVISA infection is associated with increased risk of treatment failure (van Hal and Paterson, 2011; Hu et al., 2015). Longer bacteremia and culture-positive periods lead to longer hospital stays and durations of vancomycin therapy, establishing a vicious circle in the growth of staphylococcal resistance to vancomycin (Sakoulas et al., 2006; Fong et al., 2009). Therefore, early and accurate detection of potentially non-susceptible staphylococcal strains is essential for hampering misuse of vancomycin and directing appropriate antibiotic therapy.

The Clinical and Laboratory Standards Institute defines VISA as an isolate with a minimal inhibitory concentration (MIC) of vancomycin between 4 and 8 μg per mL. The MIC of hVISA is within the susceptible range (≤ 2 μg per mL), but a subpopulation of the isolate's cells belong to a vancomycin-intermediate range (Rybak and Akins, 2001). Clinical physicians rely largely on antibiotics susceptibility tests (ASTs) to guide correct administration of antibiotics against *S. aureus* infection. However, MIC determination for *S. aureus* takes around 10 h, agar diffusion necessitates an incubation time of 18–20 h. The long turnaround time of ASTs inevitably delays accurate clinical decision-making regarding suitable antibiotics. Moreover, hVISA infection cannot be detected by routine AST methods because of its low-level vancomycin resistance and a small resistant fraction of the inoculum. hVISA can be detected by satellite colonies in the vancomycin inhibition zone and the ETest zone; it can‘t be reliably detected with automated MIC determination methods. The screening tests for hVISA are Etest glycopeptide resistance detection, the Etest macromethod, and brain heart infusion screening agar plates. These three screening tests vary in sensitivity and specificity, and single use of any one test results in poor accuracy (Satola et al., 2011). Population analysis profile–area under the curve is the gold standard of determining hVISA, but the process is cumbersome, time consuming, not commonly used in most clinical microbiology laboratories, and thus impractical for laboratory diagnosis (Chang et al., 2015).

Various proteins contribute to the resistance of *S. aureus* against vancomycin (Lin et al., 2018). The proteomic pattern of isolates can be analyzed in a rapid, comprehensive, and cost-effective manner using matrix-assisted laser desorption ionization time-of-flight (MALDI-TOF) mass spectrometry (MS) in clinical microbiology laboratories (Hrabák et al., 2013; Idelevich et al., 2017). MALDI-TOF MS produces large sets of complex data. Manual interpretation of MALDI-TOF MS data is unreliable; therefore, an informatics approach is necessary for effective and accurate interpretation. Machine learning (ML) can help automatic diagnosis and make the process less time consuming (Swan et al., 2013). The application of ML to detecting vancomycin-susceptible *S. aureus* (VSSA) in hVISA/VISA has not been widely discussed or validated (Rishishwar et al., 2014; Mather et al., 2016), although several studies have demonstrated successful application of ML in clinical practice (Wang et al., 2016; Lin et al., 2018). In the present study, we used a data processing method that facilitated the application of an ML algorithm in analysis of MALDI-TOF MS data (Wang et al., 2018). Its performance in distinguishing VSSA from hVISA/VISA was validated using nested cross-validation for a minimally biased estimation of performance (Varma and Simon, 2006; Filzmoser et al., 2009; Krstajic et al., 2014). By using the proposed ML models, we can rapidly detect hVISA/VISA and guide the use of glycopeptide for patients with MRSA infection.

## Materials and methods

### Study design

**Figure 1:**
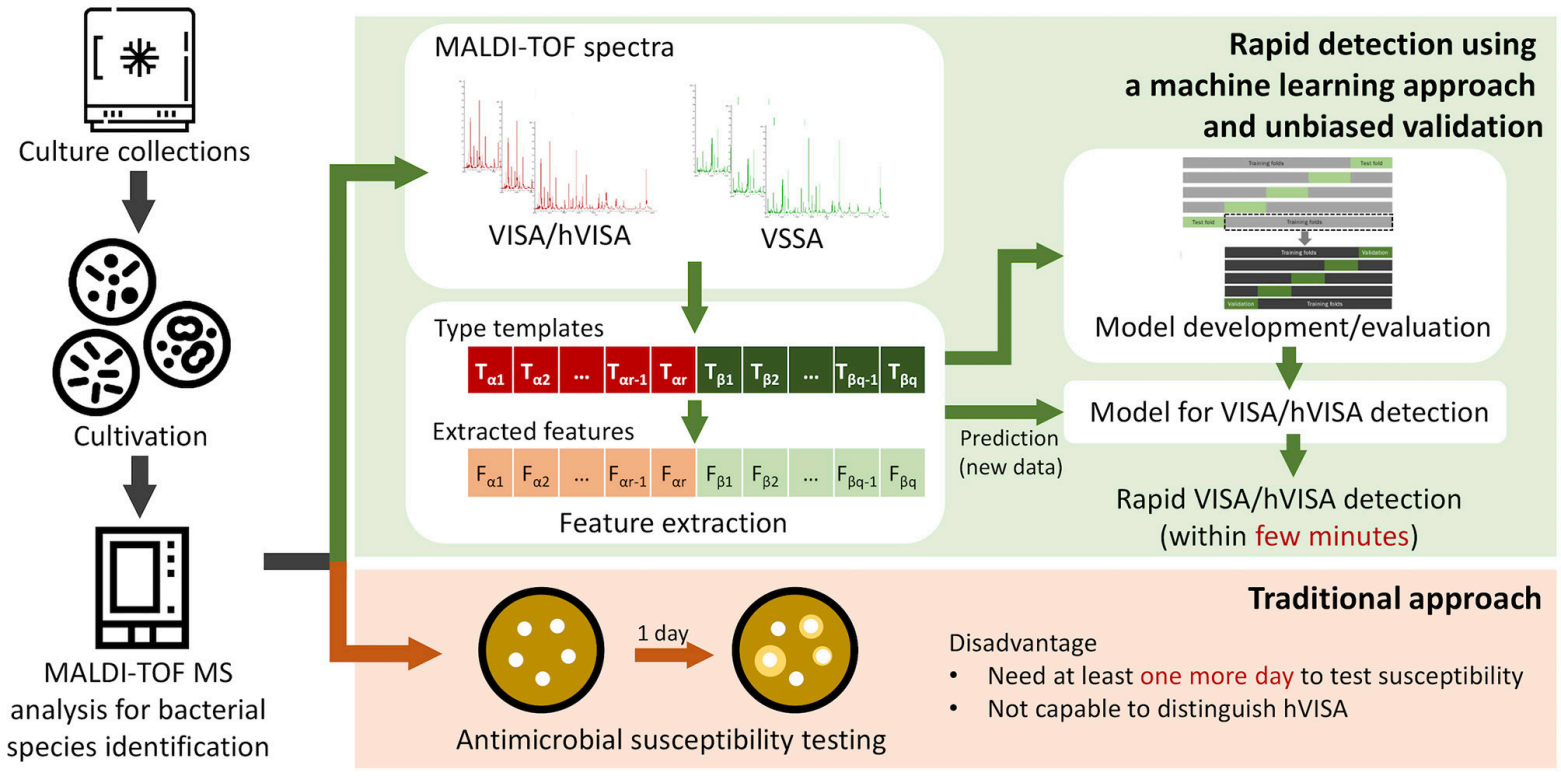
The study flow of rapid detection of hVISA based on MALDI-TOF.

The overall study flow is presented in Figure 1. MRSA isolates were cultivated from a bacterial bank (Wang et al., 2018). In the study, the 125 MRSA isolates had been collected from 2009 to 2014 at the Linkou branch of Chang Gung Memorial Hospital (CGMH), Taiwan. The specimen type was blood specimen. The MALDI-TOF MS spectra of these isolates were then obtained and relevant features selected for distinguishing VSSA from hVISA/VISA. The performance of the proposed models for rapid detection of hVISA/VISA was evaluated using a nested cross-validation approach.

### Bacterial isolates

The bacterial strains were stored at −70°C until use (Wang et al., 2018). The strains were cultured on a blood agar plate (Becton Dickinson, MD, USA) in a 5% CO_2_ incubator for 16–18 h. A colony morphology inspection, catalase test, and coagulase test were performed, and the results were in line with the characteristics of *S. aureus*. Single colonies from the blood agar plate were selected and spread onto a steel target plate (Bruker Daltonik GmbH, Bremen, Germany), followed by application of 1 mL of 70% formic acid. After being dried in ambient air, an additional 1 mL matrix solution (50% acetonitrile containing 1% α-cyano-4-hydroxycinnamic acid and 2.5% trifluoroacetic acid) was applied before analytical measurement was conducted using a Microflex LT mass spectrometer (Bruker Daltonik GmbH, Bremen, Germany). The conditions of the Microflex LT mass spectrometer were as follows: linear positive mode; accelerating voltage: +20 kV; laser frequency: 60 Hz; and laser shots per colony: up to 240. The Bruker Daltonics Bacterial Test Standard was used as an external calibration for each batch. The species of *S. aureus* was reconfirmed according to the identification results provided by Biotyper 3.1 (Bruker Daltonik GmbH, Bremen, Germany). ASTs of oxacillin were performed according to Clinical and Laboratory Standards Institute M100 S27 guideline (CLSI., 2017). A cefoxitin disc was used for testing oxacillin susceptibility. A method of multiplex polymerase chain reactions for staphylococcal cassette chromosome mec (*SCCmec*) was used for determining *SCCmec* type and detecting *mecA* to confirm MRSA (Kondo et al., 2007). The MIC of vancomycin was determined using an Etest (bioMérieux, Marcy-l'Étoile, France) according to the manufacturer's instruction. In brief, bacterial isolates were inoculated with concentration of 0.5 McFarland on Mueller Hinton agar plates (Creative Media Plate, New Taipei City, Taiwan), followed by placing vancomycin Etest strips. The MRSA isolates were screened by Etest and those with MICs ≥2–4 μg/mL were selected for modified population analysis profile–area under the curve (PAP-AUC) analyzes to be classified as either VSSA, hVISA, or VISA(Wootton, 2001). For multilocus sequence typing (MLST), seven housekeeping genes were sequenced, including carbamate kinase (*arcC*), shikimate dehydrogenase (*aroE*), glycerol kinase (*glpF*), guanylate kinase (*gmk*), phosphate acetyltransferase (*pta*), triosephosphateisomerase (*tpi*), and acetyl coenzyme A acetyltransferase (*yqiL*). The MLS typing result was determined by comparing the sequence results to the *S. aureus* MLST database (http://saureus.mlst.net/) (Enright et al., 2000).

### Analysis of MALDI-TOF MS spectra

The quality of the MS spectra was defined by the log score provided by Biotyper 3.1 (Bruker Daltonik GmbH, Bremen, Germany). MS spectra with a log score larger than 2.00 were considered acceptable quality. A spectral range from 0 to 20,000 Da was collected. Before further analysis, the MALDI-TOF MS spectra were preprocessed using Flexanalysis 3.4 (Bruker Daltonik GmbH, Bremen, Germany), as reported in a study (Wang et al., 2018). Features were extracted from the MALDI-TOF MS spectra after preprocessing. The aim of feature extraction was to standardize and facilitate the application of ML algorithms for analyzing complicated MS spectra. Feature extraction was performed on the basis of a study (Wang et al., 2018). First, type templates were constructed based on the occurrence frequency of specific peaks in the MALDI-TOF MS spectra. In the present study, the type templates of VSSA and hVISA/VISA were obtained using this approach. Features were then extracted from the MALDI-TOF MS spectra by aligning an individual spectrum onto the type templates. After the alignments, matched vectors for each type template could be obtained and an integrated vector of individual bacterial strain was generated. Supervised ML algorithms could be trained and validated according to the integrated vectors and their corresponding labels.

### Relevant feature selection

To include only the relevant features for use in the model development, we performed a feature selection step before constructing the predictive models. In each training task, a mean decrease in accuracy, obtained from the random forest algorithm (Liaw and Wiener, 2002), was employed to select the most crucial features from the training dataset. The mean decrease in accuracy was generated by measuring the effect of each feature on the accuracy of the model, permuting the values of each feature, and measuring the decrease in accuracy.

### Development of predictive models

We used random forest, a support vector machine (SVM) with a radial basis function kernel, k-nearest neighbors, and a decision tree to develop the models. Random forest is an ensemble classifier proposed by Breiman (2001) that uses random feature selection and comprises numerous classification trees. The frequency of a feature's appearance in the classification trees represents the importance of the feature. The library “randomForest” in R software (version 3.4.4, R Foundation for Statistical Computing, http://www.r-project.org/) was used for implementing the random forest classifier (Liaw and Wiener, 2002). An SVM is a data-mining method that constructs a classification model for a binary-class problem. It uses nonlinear mapping to transform the data into a higher dimension. Through appropriate nonlinear mapping to a sufficiently high dimension, data from two classes are separated by a hyperplane (Cortes and Vapnik, 1995). The library “e1071” was used for implementing the SVM classifier (Meyer et al., 2017). A simple algorithm, k-nearest neighbors stores all available cases and predicts the numerical target based on a similarity measure; it was implemented using a “class” library (Venables and Ripley, 2002). A decision tree is a recursive partitioning approach. The classification and regression trees algorithm splits each input node into two child nodes, and the same process is applied to each child node. Splitting is halted when the algorithm detects that no further gain can be made (Breiman et al., 1984). We applied the classification and regression trees algorithm to our dataset by using the “rpart” library (Therneau and Atkinson, 2018).

### Predictive model evaluation

**Figure 2:**
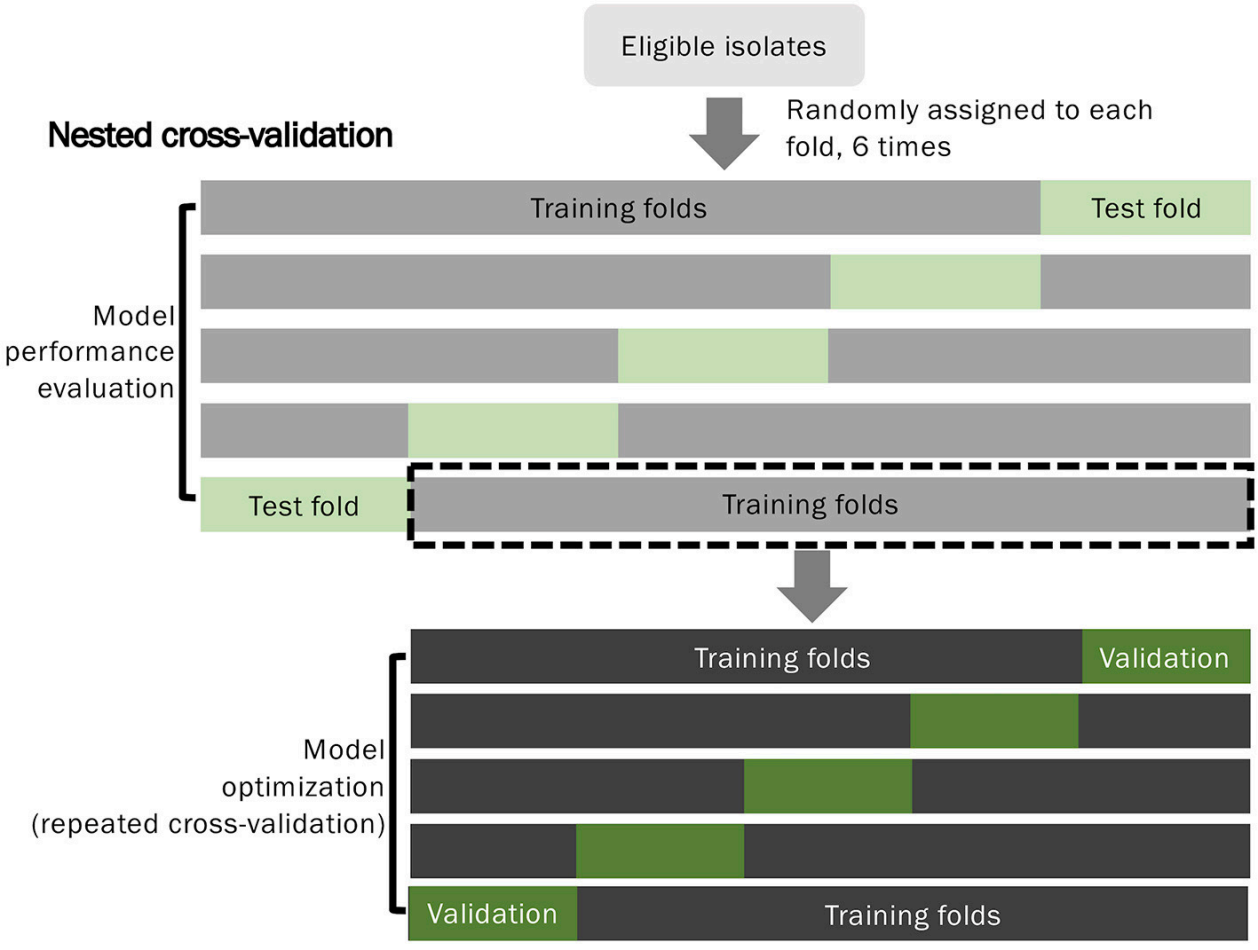
Flow diagram of the predictive model development and evaluation.

To develop predictive models for distinguishing VSSA from hVISA/VISA strains, we applied a nested 5-fold cross-validation approach to train and evaluate the models (Figure 2). In the outer 5-fold cross-validation loop, we divided the data into training (4-folds) and test (1-fold) datasets to evaluate the performance of the models with an untouched test set. In each training step in the outer fold, repeated inner 5-fold cross-validation was applied to tune and select the optimal models. The nested 5-fold cross-validation process was repeated six times to ensure our evaluation results were robust.

In each fold of outer cross-validation, we selected the features and constructed the models using data from the training set and then evaluated the performance of the models using the data in the untouched test set. The area under the receiver operating characteristic curve (AUC) was used to evaluate the performance of the models. Furthermore, we used Youden's J statistic—a single statistic that captures the performance of a dichotomous diagnostic test—to generate sensitivity and specificity for further analysis of prediction performance.

### Statistical analysis

The Mann–Whitney *U*-test was used to analyze MALDI-TOF MS spectra peak characteristics. We performed analysis of variance (ANOVA) and Tukey honestly significant difference *post-hoc* analyses on the AUC values of the predictive models. All analyses were performed using the R software. All statistical tests were two-sided, and statistical significance was defined as *p* < 0.05.

### Data availability

The raw data supporting the conclusions of this manuscript will be made available by the authors, without undue reservation, to any qualified researcher.

## Results

### MALDI-TOF MS spectra of MRSA isolates

A total of 125 MRSA isolates, namely 35 hVISA/VISA and 90 VSSA strains, were used to develop the proposed predictive models. For each isolate, 127 peaks were extracted from a mass spectrum. The peak characteristics and their intensities for these MRSA isolates are presented in Supplementary Table 1. Among these peaks, the intensities of 13 peaks were differed significantly between the hVISA/VISA and VSSA strains (Table 1).

**Table 1:** MALDI-TOF MS spectra peak characteristics that their intensities differed significantly between the hVISA/VISA and VSSA strains.

**Peak, m/z**	**Intensity**		***P*-value^d^**
	**hVISA^a^/VISA^b^ strains (median [IQR])**	**VSSA^c^ strains (median [IQR])**	
118	12.97 [11.58, 14.32]	11.39 [0.00, 13.20]	0.005
119	12.97 [11.58, 14.32]	11.41 [0.00, 13.23]	0.005
680	0.00 [0.00, 0.00]	0.00 [0.00, 0.00]	0.006
852	0.00 [0.00, 12.26]	12.37 [0.00, 13.46]	0.005
948	0.00 [0.00, 11.87]	0.00 [0.00, 0.00]	0.006
1132	0.00 [0.00, 11.42]	0.00 [0.00, 0.00]	<0.001
1266	0.00 [0.00, 11.62]	0.00 [0.00, 0.00]	0.009
2429	12.37 [0.00, 13.31]	0.00 [0.00, 12.44]	0.004
2895	0.00 [0.00, 11.82]	0.00 [0.00, 0.00]	<0.001
3176	10.65 [0.00, 11.18]	0.00 [0.00, 10.64]	0.001
6351	10.86 [10.58, 11.15]	10.62 [2.51, 10.94]	0.009
6591	10.54 [10.10, 11.02]	0.00 [0.00, 10.70]	<0.001
9625	12.66 [12.32, 12.92]	12.30 [11.77, 12.81]	0.01

a *Heterogeneous Vancomycin-intermediate S. aureus*.

b *Vancomycin-intermediate S. aureus*.

c *Vancomycin-susceptible S. aureus*.

d *Mann–Whitney U test*.

### Relevant features for distinguishing VSSA from hVISA/VISA

**Figure 3:**
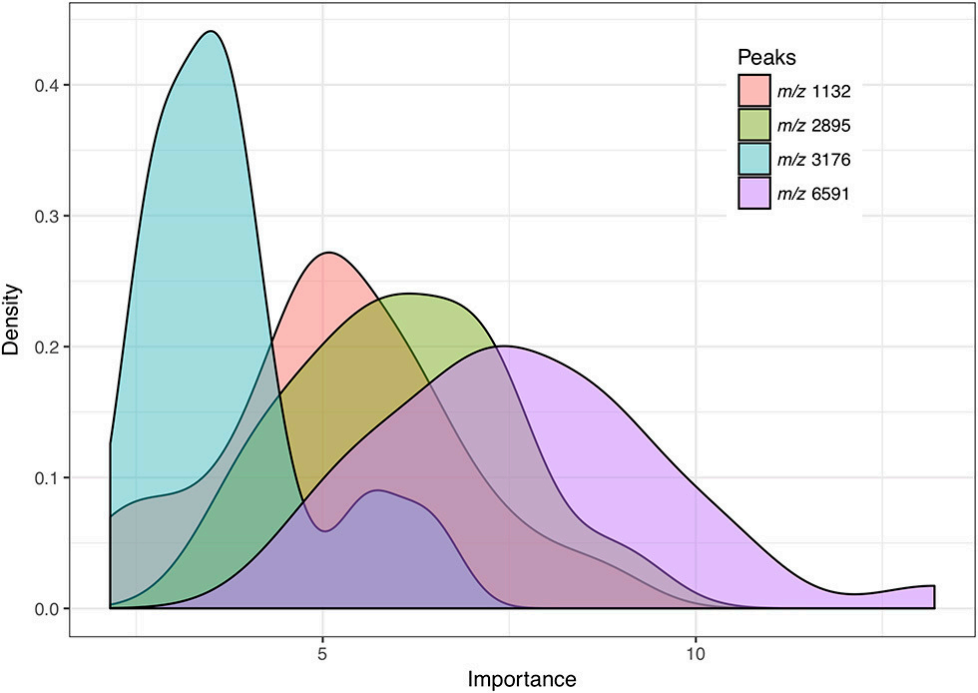
Distribution of importance based on kernel density estimation of peak features selected by more than 90% of the predictive models. Importance: z-score of mean decrease in accuracy obtained from the random forest algorithm.

We defined relevant peak features as peaks with importance greater than 1.9 based on the random forest algorithm results. The importance was defined as z-score of mean decrease in accuracy obtained from the random forest algorithm (Liaw and Wiener, 2002). Among the 109 relevant features selected from 30 feature selection results based on repeated nested 5-fold cross-validation (Supplementary Table 2), four peak features were selected in more than 90% of the models. Figure 3 shows the distribution based on kernel density estimation of the importance of four peak features. The peak at m/z 6591 was selected as a relevant feature in all the training tasks (*n* = 30) and identified as the most crucial feature for distinguishing VSSA from hVISA/VISA.

### Performance of the predictive models

**Figure 4:**
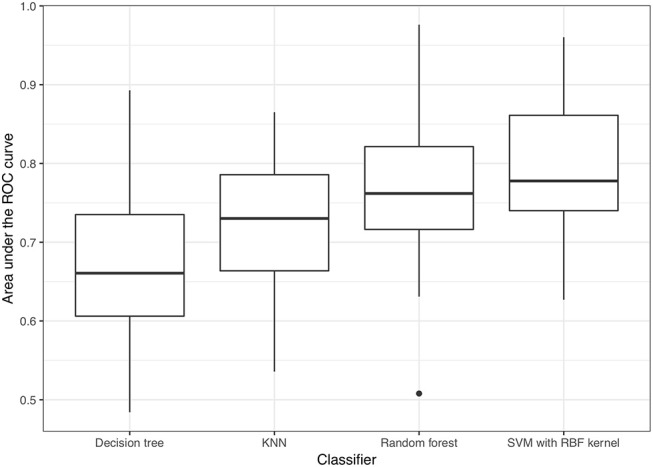
Performance of predictive models for distinguishing VSSA from hVISA/VISA isolates. KNN: k-nearest neighbor; SVM, support vector machine; RBF kernel, radial basis function kernel.

**Figure 5:**
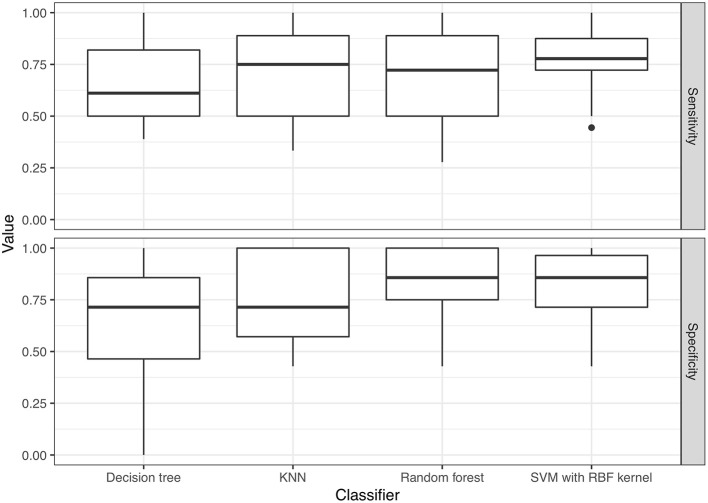
Sensitivity and specificity of the predictive models, calculated based on the maximum value of Youden's J statistic. KNN, k-nearest neighbor; SVM, support vector machine; RBF kernel, radial basis function kernel.

Regarding model performance for distinguishing VSSA strains from hVISA/VISA strains among the MRSA isolates, the optimal predictive model for the test set was the model constructed using the SVM classifier with a radial basis function kernel and with AUC = 0.790. The model constructed using the random forest algorithm had similar performance, with AUC = 0.763 (*p* = 0.30). The AUCs for the models constructed using k-nearest neighbors and a decision tree were 0.722 and 0.668, respectively (Figure 4), which were lower than those of the optimal predictive models (*p* < 0.01). Based on the maximum value of Youden's J statistic, the average sensitivity and specificity of the SVM classifier were 0.770 and 0.814, respectively (Figure 5). The validation results of all the classifiers using nested 5-fold cross-validation, repeated 6 times, were presented in Supplementary Table 3.

## Discussion

In the present study, we demonstrated that the ML-based approach can successfully distinguish VSSA from hVISA/VISA on the basis of MALDI-TOF MS data. The preliminary AST obtained from the ML-based approach can yield an accurate and rapid administration of correct antibiotics against MRSA infection.

To distinguish hVISA/VISA from VSSA, a local ML model can be established using the proposed strategy, and local clinical microbiologists can easily acquire ML models that adequately fit their own population. The prevalence of hVISA differs among countries and areas (Zhang et al., 2015). Up to 50% of isolates reported as susceptible to vancomycin can harbor hVISA clones (Horne et al., 2009). Moreover, the composition of isolates potentially varies among different areas. Consequently, a localized ML model trained by locally relevant data would offer superior performance to a general model. The strengths of the ML models proposed in this study are their rapidness and low cost. A vancomycin susceptibility test report could be obtained using MALDI-TOF MS alone without other testing methods. Clinical microbiologists could provide preliminary but accurate vancomycin susceptibility days prior to PAP-AUC, which is regarded as time-consuming and expensive. Although various other hVISA screening tools have been developed, these methods are typically culture-dependent and require a long incubation time (Riederer et al., 2011; van Hal et al., 2011). Moreover, the ML models do not require additional hVISA screening tests such as glycopeptide resistance detection, the Etest macromethod, or brain heart infusion screening agar plate screening to report hVISA. Therefore, the cost of diagnosis could be considerably decreased. The MALDI-TOF MS used in this study was performed with direct deposit of bacteria onto a steel plate rather than extracting it and placing in a tube. Direct deposition is used in routine practice because it is rapid and not labor-intensive. We used typical sample processing methods so that the proposed ML models could cope with MALDI-TOF MS data in real-world applications.

Incorporating ML algorithms into prediction of antibiotics susceptibility is a promising application of ML. However, its associated issues have not been widely addressed. One study reported detection of hVISA/VISA using ML to analyze MALDI-TOF MS data. The authors used an SVM and correctly identified 100% of VISA and 97% of VSSA isolates with an overall classification accuracy of 98% (Mather et al., 2016). The performance was promising, and the authors also demonstrated that the performance did not result from the specific composition of the bacterial isolates (Mather et al., 2016). However, bias may still have existed, because only 21 VISA, 21 hVISA, and 38 VSSA isolates were used in the study. Moreover, the feature selection (essential peak selection) and model optimization steps appeared to be conducted within all the datasets, not within an independent training dataset, which may have resulted in overfitting and thus perfect performance. Another study also detailed a promising model with 99% sensitivity and 88% specificity for classifying VSSA, VISA, and hVISA (Asakura et al., 2018). The study provided a graphical user interface with fully public release code, which could truly benefit health care and research teams. However, due to the study's selection of multiple colonies from one hVISA strain and the use of leave-one-out validation, the model was also likely to be overfitted. Given the high fidelity of MALDI-TOF MS (Croxatto et al., 2012), we did not replicate each isolate by performing multiple MALDI-TOF analyses as did by other study (Asakura et al., 2018). Oversampling by direct replicating the isolates may result in overfitting bias (Kubat and Matwin, 1997; Kegelmeyer et al., 2002; Guo et al., 2008). By contrast, we used nested cross-validation to avoid overfitting. The feature selection step and model tuning were conducted within an independent training dataset in each iteration (Figure 2). Consequently, the selected feature compositions were different (Supplementary Table 2). The importance of the features could be determined by their frequency of occurrence in the nested cross-validation. As shown in Supplementary Table 2 and Figure 3, the ions at m/z 1132, 2895, 3176, and 6591 were selected as the essential peaks and were selected in more than 90% of the predictive models. The ions at m/z 6887 and 9625, were selected with moderate frequency (Supplementary Table 2), whereas the ion at m/z 3006 was selected as an essential peak in only a few iterations (Supplementary Table 2). The results indicated the necessity of selecting features using an independent training dataset. A peak may be mistaken as an essential peak when iteration is not used. We confirmed the importance of characteristic peaks by using nested cross-validation. In this work, we analyzed the region from 0 to 20000 m/z because we did not presume that a characteristic peak cannot be found under 2000 m/z. We just included all the data and discover meaningful information by a data mining technique (i.e., feature selection process in this study). In most of studies, region 2000 to 20000 m/z was used for analysis, and some irregular peaks from the agar medium may show up within the region below 2000 m/z. To avoid an irregular peak being selected as a characteristic peak, random forest algorithm was applied to estimate the importance of each peak in discriminating VSSA from hVISA/VISA, under the scheme of nested cross validation (Figure 2). Characteristic peaks would be selected through the unbiased method.

The ions at m/z 1132, 2895, 3176, and 6591 were the crucial features in distinguishing VSSA from hVISA/VISA in the present study (Supplementary Table 2 and Figure 3). Lu et al. reported that the ions at m/z 1835 and 1863 were characteristic peaks for hVISA and VISA (Lu et al., 2012). However, Mather et al. revealed that ions at m/z 4540 and 8258 were characteristic for VISA and VSSA, respectively (Mather et al., 2016). This discordance may be due to several reasons. First, the bacterial isolates were acquired from different locations and at different times. Second, the extraction methods were also different; tube extraction was used in these two studies (Lu et al., 2012; Mather et al., 2016), whereas we used direct deposition, which is the method used in routine practice. Third, the aforementioned difference in the methods of selecting essential peaks could also account for the discordance. In the previous studies, the characteristic peaks were selected on the basis of either descriptive statistics (Lu et al., 2012) or multiple regression (Mather et al., 2016). By contrast, we selected characteristic peaks by using random forest feature importance and confirmed the importance of the peaks in multiple iterations. In the present study, the ion at m/z 6591 was detected in 85.7 and 41.1% of the hVISA/VISA and VSSA groups, respectively. Previous studies have demonstrated m/z 6591 as a characteristic peak of clonal complex 8 (CC8) MRSA isolates (Wolters et al., 2011; Boggs et al., 2012; Josten et al., 2013; Camoez et al., 2016). In the first study conducted by Wolters et al. a model was demonstrated with the ability to discriminate five major CCs (CC5, CC8, CC22, CC30, and CC45) by using 13 peaks, including m/z 6591, which appeared to be specific to CC8 isolates (Wolters et al., 2011). In another study, m/z 6591 was adopted as one of the three peaks of a classifier constructed from 47 USA300/CC8 and 77 non-USA300 MRSA isolates. The classifier had an 87.9% overall accuracy on a validation dataset (Boggs et al., 2012). In 2013, Josten et al. analyzed the peak pattern of 401 MRSA and MSSA strains, revealing that the peak protein at m/z 6592 provided a sensitivity of 0.889 and specificity of 0.996 for CC8 (Josten et al., 2013). In 2016, a supervised neural network model constructed by Camoez et al. on the basis of data covering a 20 years period suggested m/z 6591.84 as a unique biomarker of CC8 isolates (Camoez et al., 2016). In our data, m/z 6591 was also noted in 56 of 62 CC8 and ST239 strains (90.3%). Our results are consistent with those of previous studies conducted in Europe and the United States, which suggests that despite geographical and racial diversity, peak protein m/z 6591 can provide valuable classification information regarding MRSA in Asian populations. Although ions at m/z 1132, 2895, and 3176 were also selected as informative features in the present study, the significance and relation of these features in the resistance of VISA and hVISA have not yet been reported.

This study had several limitations. First, bacterial composition affected the performance of the ML models. The performance of ML can be compromised by a complex bacterial composition. In this study, the bacterial composition of the isolates was analyzed using multilocus sequence and *SCCmec* typing. The bacterial composition results revealed a non-restricted bacterial distribution, for which classification problems are not generally simple (Supplementary Figure 1). The ML models and results may not be generalized directly to other countries or areas. The MLS type of most MRSA isolates in this study are ST239 (62/125), followed by ST5 and ST59 (Supplementary Figure 1). This is the distinct composition of MRSA isolates in Taiwan (Sheng et al., 2009), and the characteristic peaks and the models created based on the cohort may be only used for the population in this region. In this study, we demonstrated a ML-based methodology for detecting hVISA/VISA. Through using the workflow proposed in this study, other clinical microbiology laboratories could obtain their own ML models specific for detecting hVISA/VISA in their region. We did not aim to and may not possibly generalize the ML models but we proposed a methodology which may help others generating a specific model fitting their populations more properly than do a generalized model. Second, the ML performance reported in this study is not as high as that reported in other studies that evaluated model performance using leave-one-out cross-validation (Rishishwar et al., 2014; Mather et al., 2016). This lower performance may have resulted from the stricter validation method applied in the present study. We used direct deposition instead of in-tube extraction. The direct deposition method offers a rapid turnaround time and requires less labor; however, the reproducibility and quality of MALDI-TOF MS data may be compromised (Goldstein et al., 2013; Mather et al., 2016). Compromised MALDI-TOF data may reduce ML model performance because non-susceptible *S. aureus* is relatively rare (10^−5^-10^−6^) in hVISA (van Hal and Paterson, 2011); more sensitive MALDI-TOF data could facilitate detection of subtle changes during MS. Third, although the performance of the ML models was validated using a minimally biased method, the models should undergo external validation in other Taiwanese institutes. Fourth, the primary aim of this study is to demonstrate and validate an unbiased methodology to detect hVISA/VISA by analyzing MALDI-TOF MS spectra through a ML-based approach. We focus more on the aspect of clinical application in this work. The validated ML model is ready to be used in our clinical practice and hopefully the proposed method can help generate clinically useful ML model in other local clinical microbiology laboratories. Besides, identifying protein/peptide behind the peaks is essential for understanding the causative proteins/mechanisms for vancomycin resistance, which is worthy further investigation in the future. In general, the present study successfully demonstrated the use of an ML approach for detecting hVISA/VISA. The negative predictive value of detecting vancomycin-non-susceptible *S. aureus* was 0.9695 when the prevalence of hVISA was 10%. Additionally, the absolute reduction of risk of administering inadequate glycopeptide dose in treating vancomycin-non-susceptible *S. aureus* was 0.0695 under the prevalence setting.

In conclusion, the proposed ML models, validated by a robust model evaluation method, successfully distinguished emerging superbugs (hVISA/VISA) from VSSA, which cannot be detected in most clinical microbiology laboratories. By utilizing cost-effective MALDI-TOF and ML technologies, providers have the opportunity to offer rapid and accurate treatment for MRSA.

## Author contributions

H-YW and Y-JT had full access to all the data in the study and take responsibility for the integrity of the data, and the accuracy of the data analysis. H-YW and Y-JT analyzed/interpreted the data, performed experiments, designed the study, and wrote the paper. C-HC, T-YL, J-TH, T-PL, and J-JL reviewed/edited the manuscript for important intellectual content and provided administrative, technical, or material support. Y-JT and J-JL obtained funding and supervised the study.

### Conflict of interest statement

The authors declare that the research was conducted in the absence of any commercial or financial relationships that could be construed as a potential conflict of interest.
