# Supplementary material for: Rapid Detection of Heterogeneous Vancomycin-Intermediate Staphylococcus aureus Based on Matrix-Assisted Laser Desorption Ionization Time-of-Flight: Using a Machine Learning Approach and Unbiased Validation
Source: Front Microbiol. 2018 Oct 11;9:2393. doi: 10.3389/fmicb.2018.02393 (PMC6193097; doi:10.3389/fmicb.2018.02393)
Supplement: Supplementary file 1 [file Table_1.DOCX]

**Supplementary Table 1**. MALDI-TOF MS spectra peak characteristics

| Peak, m/z  (median [IQR]) | Intensity | | P-value^d^ |
| --- | --- | --- | --- |
|  | **hVISA^a^/VISA^b^ strains**  **(median [IQR])** | **VSSA^c^ strains**  **(median [IQR])** |  |
| 23 | 0.00 [0.00, 11.01] | 0.00 [0.00, 12.01] | 0.569 |
| 47 | 0.00 [0.00, 0.00] | 0.00 [0.00, 0.00] | 0.828 |
| 48 | 0.00 [0.00, 11.25] | 0.00 [0.00, 0.00] | 0.384 |
| 65 | 0.00 [0.00, 13.66] | 0.00 [0.00, 7.69] | 0.08 |
| 102 | 0.00 [0.00, 10.78] | 0.00 [0.00, 11.03] | 0.839 |
| 108 | 0.00 [0.00, 0.00] | 0.00 [0.00, 0.00] | 0.306 |
| 118 | 12.97 [11.58, 14.32] | 11.39 [0.00, 13.20] | 0.005 |
| 119 | 12.97 [11.58, 14.32] | 11.41 [0.00, 13.23] | 0.005 |
| 120 | 12.94 [11.49, 14.32] | 11.87 [0.00, 13.23] | 0.019 |
| 121 | 12.94 [11.49, 14.32] | 12.01 [2.51, 13.17] | 0.057 |
| 132 | 11.57 [0.00, 13.32] | 12.67 [0.00, 13.62] | 0.147 |
| 137 | 12.50 [11.29, 13.77] | 12.87 [11.66, 14.16] | 0.664 |
| 139 | 12.38 [11.46, 13.60] | 12.33 [11.18, 13.93] | 0.73 |
| 140 | 12.31 [11.57, 13.60] | 12.24 [10.66, 13.34] | 0.341 |
| 149 | 11.78 [0.00, 13.00] | 12.20 [0.00, 13.32] | 0.291 |
| 150 | 11.78 [0.00, 13.23] | 12.58 [0.00, 13.43] | 0.335 |
| 156 | 13.32 [12.44, 14.09] | 13.36 [12.52, 14.32] | 0.593 |
| 158 | 13.32 [12.43, 14.23] | 13.32 [12.41, 14.32] | 0.982 |
| 177 | 13.32 [0.00, 13.82] | 13.32 [0.00, 14.33] | 0.118 |
| 183 | 0.00 [0.00, 13.12] | 0.00 [0.00, 13.23] | 0.236 |
| 198 | 13.32 [12.61, 14.32] | 13.32 [12.23, 14.32] | 0.457 |
| 200 | 12.84 [5.24, 14.11] | 12.53 [0.00, 13.80] | 0.616 |
| 214 | 12.40 [0.00, 13.26] | 12.50 [0.00, 13.50] | 0.47 |
| 215 | 12.68 [0.00, 13.32] | 13.14 [10.16, 14.08] | 0.155 |
| 227 | 12.04 [0.00, 13.31] | 0.00 [0.00, 13.13] | 0.144 |
| 230 | 12.64 [0.00, 13.32] | 0.00 [0.00, 13.24] | 0.149 |
| 233 | 13.10 [11.64, 13.55] | 12.60 [0.00, 13.67] | 0.406 |
| 236 | 12.64 [10.82, 13.84] | 12.63 [10.64, 13.90] | 0.947 |
| 242 | 11.33 [0.00, 13.11] | 0.00 [0.00, 12.67] | 0.255 |
| 253 | 12.87 [12.21, 13.67] | 13.06 [12.02, 13.78] | 0.978 |
| 256 | 12.87 [12.16, 13.67] | 13.06 [11.64, 13.78] | 0.971 |
| 266 | 0.00 [0.00, 13.12] | 0.00 [0.00, 0.00] | 0.314 |
| 276 | 12.35 [0.00, 14.32] | 12.01 [0.00, 13.62] | 0.266 |
| 278 | 13.07 [11.24, 14.32] | 12.53 [0.00, 14.01] | 0.238 |
| 280 | 13.04 [11.36, 14.32] | 12.84 [10.49, 14.07] | 0.354 |
| 292 | 12.10 [0.00, 13.32] | 13.09 [10.71, 14.01] | 0.153 |
| 293 | 12.10 [0.00, 13.32] | 13.04 [0.00, 14.01] | 0.23 |
| 305 | 13.32 [12.31, 14.09] | 13.18 [12.00, 14.26] | 0.741 |
| 306 | 13.32 [12.31, 14.09] | 13.10 [11.95, 14.18] | 0.516 |
| 322 | 14.13 [13.32, 14.90] | 14.23 [12.91, 14.59] | 0.46 |
| 323 | 14.13 [13.32, 14.90] | 14.23 [12.91, 14.59] | 0.46 |
| 324 | 13.32 [0.00, 14.90] | 13.32 [0.00, 14.56] | 0.474 |
| 335 | 0.00 [0.00, 12.36] | 0.00 [0.00, 13.09] | 0.351 |
| 349 | 12.80 [11.63, 13.47] | 13.02 [2.56, 13.93] | 0.956 |
| 365 | 12.62 [0.00, 13.32] | 13.32 [0.00, 14.32] | 0.036 |
| 366 | 12.62 [0.00, 13.32] | 13.32 [0.00, 14.32] | 0.034 |
| 367 | 12.62 [0.00, 13.32] | 13.32 [0.00, 14.32] | 0.06 |
| 379 | 0.00 [0.00, 13.32] | 0.00 [0.00, 8.27] | 0.112 |
| 389 | 12.27 [5.59, 13.59] | 12.37 [0.00, 13.54] | 0.756 |
| 390 | 12.27 [5.59, 13.59] | 12.37 [0.00, 13.54] | 0.756 |
| 429 | 12.06 [0.00, 13.27] | 12.40 [0.00, 13.32] | 0.31 |
| 441 | 0.00 [0.00, 0.00] | 0.00 [0.00, 0.00] | 0.736 |
| 480 | 0.00 [0.00, 12.15] | 0.00 [0.00, 0.00] | 0.021 |
| 496 | 10.97 [0.00, 12.73] | 0.00 [0.00, 12.33] | 0.557 |
| 497 | 11.34 [0.00, 12.86] | 10.62 [0.00, 12.65] | 0.36 |
| 512 | 11.58 [0.00, 12.24] | 11.95 [0.00, 13.00] | 0.161 |
| 513 | 0.00 [0.00, 11.95] | 11.13 [0.00, 12.69] | 0.194 |
| 525 | 11.82 [0.00, 13.10] | 0.00 [0.00, 12.78] | 0.209 |
| 527 | 12.86 [12.05, 13.46] | 12.65 [11.39, 13.93] | 0.877 |
| 529 | 12.43 [0.00, 13.39] | 12.06 [0.00, 13.49] | 0.767 |
| 530 | 12.43 [0.00, 13.39] | 12.06 [0.00, 13.49] | 0.767 |
| 540 | 0.00 [0.00, 12.99] | 0.00 [0.00, 12.34] | 0.981 |
| 557 | 0.00 [0.00, 12.43] | 11.72 [0.00, 13.49] | 0.084 |
| 578 | 0.00 [0.00, 12.36] | 0.00 [0.00, 0.00] | 0.042 |
| 606 | 0.00 [0.00, 12.23] | 5.38 [0.00, 13.09] | 0.317 |
| 615 | 0.00 [0.00, 0.00] | 0.00 [0.00, 0.00] | 0.822 |
| 680 | 0.00 [0.00, 0.00] | 0.00 [0.00, 0.00] | 0.006 |
| 815 | 0.00 [0.00, 14.28] | 0.00 [0.00, 0.00] | 0.059 |
| 834 | 13.32 [5.77, 13.59] | 13.32 [2.86, 14.27] | 0.351 |
| 852 | 0.00 [0.00, 12.26] | 12.37 [0.00, 13.46] | 0.005 |
| 860 | 0.00 [0.00, 0.00] | 0.00 [0.00, 0.00] | 0.871 |
| 948 | 0.00 [0.00, 11.87] | 0.00 [0.00, 0.00] | 0.006 |
| 964 | 0.00 [0.00, 11.77] | 0.00 [0.00, 11.90] | 0.549 |
| 1132 | 0.00 [0.00, 11.42] | 0.00 [0.00, 0.00] | <0.001 |
| 1145 | 0.00 [0.00, 11.02] | 0.00 [0.00, 11.30] | 0.437 |
| 1266 | 0.00 [0.00, 11.62] | 0.00 [0.00, 0.00] | 0.009 |
| 1277 | 10.28 [0.00, 12.46] | 5.13 [0.00, 12.16] | 0.476 |
| 1278 | 11.89 [0.00, 12.68] | 11.14 [0.00, 12.52] | 0.233 |
| 2241 | 10.38 [0.00, 12.47] | 11.19 [0.00, 12.18] | 0.736 |
| 2242 | 10.71 [0.00, 12.47] | 11.33 [0.00, 12.24] | 0.704 |
| 2285 | 11.84 [0.00, 13.29] | 12.37 [0.00, 13.54] | 0.261 |
| 2303 | 12.61 [0.00, 13.32] | 13.01 [0.00, 14.08] | 0.221 |
| 2320 | 0.00 [0.00, 12.38] | 11.51 [0.00, 12.92] | 0.074 |
| 2413 | 12.70 [0.00, 13.32] | 0.00 [0.00, 13.30] | 0.046 |
| 2429 | 12.37 [0.00, 13.31] | 0.00 [0.00, 12.44] | 0.004 |
| 2545 | 0.00 [0.00, 11.18] | 0.00 [0.00, 12.21] | 0.242 |
| 2634 | 12.49 [10.16, 13.31] | 13.01 [11.11, 14.08] | 0.162 |
| 2650 | 12.24 [11.72, 12.74] | 12.35 [11.86, 12.81] | 0.353 |
| 2761 | 11.37 [10.95, 12.06] | 11.49 [0.00, 12.14] | 0.634 |
| 2877 | 11.51 [0.00, 13.10] | 0.00 [0.00, 11.51] | 0.016 |
| 2895 | 0.00 [0.00, 11.82] | 0.00 [0.00, 0.00] | <0.001 |
| 2977 | 11.46 [0.00, 12.47] | 0.00 [0.00, 12.23] | 0.115 |
| 3006 | 13.11 [11.02, 13.46] | 12.63 [11.17, 13.32] | 0.531 |
| 3007 | 13.11 [11.02, 13.46] | 12.63 [11.17, 13.32] | 0.531 |
| 3039 | 10.32 [0.00, 12.84] | 11.91 [0.00, 13.32] | 0.073 |
| 3054 | 10.92 [0.00, 12.15] | 10.73 [0.00, 12.28] | 0.831 |
| 3176 | 10.65 [0.00, 11.18] | 0.00 [0.00, 10.64] | 0.001 |
| 3209 | 11.77 [11.37, 11.99] | 11.61 [11.30, 11.99] | 0.432 |
| 3421 | 11.26 [11.13, 11.67] | 11.29 [10.85, 11.65] | 0.603 |
| 3444 | 12.55 [12.20, 13.07] | 12.74 [12.03, 13.21] | 0.965 |
| 3783 | 11.00 [10.72, 11.41] | 11.01 [10.52, 11.34] | 0.544 |
| 4045 | 10.88 [10.53, 11.24] | 10.74 [2.42, 11.23] | 0.153 |
| 4075 | 9.94 [0.00, 10.58] | 0.00 [0.00, 10.09] | 0.029 |
| 4305 | 12.14 [11.89, 12.35] | 11.87 [11.33, 12.26] | 0.022 |
| 4446 | 11.13 [10.83, 11.25] | 10.98 [10.59, 11.37] | 0.319 |
| 4590 | 11.12 [10.74, 11.43] | 10.91 [10.60, 11.31] | 0.222 |
| 4813 | 13.32 [13.22, 13.61] | 13.24 [12.89, 13.62] | 0.06 |
| 4827 | 10.90 [0.00, 11.66] | 11.21 [0.00, 11.73] | 0.323 |
| 4862 | 0.00 [0.00, 10.81] | 0.00 [0.00, 10.27] | 0.149 |
| 5031 | 11.85 [11.50, 12.11] | 11.61 [11.26, 11.95] | 0.05 |
| 5032 | 11.85 [11.49, 12.11] | 11.56 [11.22, 11.95] | 0.059 |
| 5052 | 10.78 [10.51, 11.19] | 10.76 [0.00, 11.18] | 0.288 |
| 5240 | 11.25 [10.64, 11.60] | 11.09 [10.71, 11.46] | 0.622 |
| 5302 | 11.88 [11.58, 12.35] | 11.87 [11.34, 12.33] | 0.409 |
| 5303 | 11.88 [11.56, 12.32] | 11.81 [11.26, 12.31] | 0.448 |
| 5524 | 12.80 [12.46, 13.11] | 12.72 [12.17, 13.17] | 0.361 |
| 6351 | 10.86 [10.58, 11.15] | 10.62 [2.51, 10.94] | 0.009 |
| 6422 | 11.37 [11.08, 11.71] | 11.26 [10.86, 11.54] | 0.181 |
| 6591 | 10.54 [10.10, 11.02] | 0.00 [0.00, 10.70] | <0.001 |
| 6612 | 10.38 [10.05, 10.90] | 10.27 [0.00, 10.73] | 0.126 |
| 6815 | 10.97 [10.75, 11.29] | 11.04 [10.63, 11.39] | 0.869 |
| 6816 | 10.97 [10.75, 11.29] | 11.03 [10.55, 11.39] | 0.996 |
| 6843 | 11.46 [11.29, 11.72] | 11.42 [11.16, 11.66] | 0.397 |
| 6887 | 13.29 [13.08, 13.46] | 13.29 [12.72, 13.72] | 0.499 |
| 7565 | 10.74 [10.37, 11.18] | 10.63 [10.20, 11.07] | 0.147 |
| 8148 | 10.02 [9.58, 10.60] | 9.77 [0.00, 10.33] | 0.036 |
| 9625 | 12.66 [12.32, 12.92] | 12.30 [11.77, 12.81] | 0.01 |

**^a^** Heterogeneous Vancomycin-intermediate *S. aureus*. **^b^** Vancomycin-intermediate *S. aureus*. **^c^** Vancomycin-susceptible *S. aureus*. **^d^** Mann–Whitney U test.
