# Supplementary material for: Rapid Detection of Heterogeneous Vancomycin-Intermediate Staphylococcus aureus Based on Matrix-Assisted Laser Desorption Ionization Time-of-Flight: Using a Machine Learning Approach and Unbiased Validation
Source: Front Microbiol. 2018 Oct 11;9:2393. doi: 10.3389/fmicb.2018.02393 (PMC6193097; doi:10.3389/fmicb.2018.02393)
Supplement: Supplementary file 2 [file Table_2.DOCX]

**Supplementary Table 2**. Relevant features for distinguish VSSA from hVISA/VISA

| Peak, m/z | Selected times | Feature importance, mean (SD) |
| --- | --- | --- |
| 6591 | 30 | 7.71 (1.92) |
| 2895 | 29 | 6.07 (1.41) |
| 1132 | 27 | 5.19 (1.61) |
| 3176 | 27 | 3.75 (1.11) |
| 118 | 26 | 3.57 (1.28) |
| 119 | 26 | 3.33 (1.22) |
| 852 | 25 | 4.26 (1.35) |
| 1266 | 24 | 3.48 (0.9) |
| 1277 | 24 | 4.12 (1.33) |
| 512 | 24 | 3.03 (0.65) |
| 4075 | 23 | 3.25 (0.86) |
| 2877 | 21 | 3.56 (1.21) |
| 2241 | 20 | 2.88 (0.74) |
| 9625 | 20 | 3.38 (1.1) |
| 5032 | 19 | 2.89 (0.84) |
| 4813 | 18 | 3.25 (0.9) |
| 948 | 18 | 2.97 (1.3) |
| 365 | 17 | 2.66 (0.51) |
| 5031 | 17 | 3.02 (0.91) |
| 6351 | 17 | 3.11 (0.65) |
| 4446 | 16 | 2.76 (0.63) |
| 6887 | 16 | 3.34 (1.2) |
| 1145 | 15 | 3.48 (0.93) |
| 120 | 15 | 2.76 (0.54) |
| 1278 | 15 | 3.01 (0.59) |
| 2429 | 15 | 3.3 (1.02) |
| 366 | 15 | 2.81 (0.47) |
| 4305 | 15 | 2.64 (0.56) |
| 121 | 13 | 2.73 (0.58) |
| 2242 | 13 | 3.12 (0.71) |
| 367 | 13 | 2.97 (0.63) |
| 680 | 13 | 3.25 (1.05) |
| 177 | 10 | 2.58 (0.49) |
| 2634 | 10 | 2.76 (0.72) |
| 349 | 9 | 2.18 (0.26) |
| 6422 | 9 | 2.52 (0.6) |
| 5524 | 8 | 2.7 (0.94) |
| 215 | 7 | 2.41 (0.5) |
| 3039 | 7 | 2.29 (0.28) |
| 3444 | 7 | 3.02 (0.73) |
| 4827 | 7 | 2.68 (0.52) |
| 480 | 6 | 2.52 (0.61) |
| 292 | 5 | 2.42 (0.3) |
| 324 | 5 | 2.22 (0.29) |
| 497 | 5 | 2.29 (0.33) |
| 513 | 5 | 2.92 (0.52) |
| 6816 | 5 | 2.53 (0.46) |
| 108 | 4 | 2.82 (0.74) |
| 137 | 4 | 2.31 (0.25) |
| 140 | 4 | 2.5 (0.86) |
| 183 | 4 | 2.13 (0.17) |
| 2285 | 4 | 2.45 (0.42) |
| 230 | 4 | 2.39 (0.5) |
| 233 | 4 | 2.66 (0.49) |
| 2413 | 4 | 2.63 (0.79) |
| 2545 | 4 | 2.48 (0.31) |
| 2650 | 4 | 2.3 (0.18) |
| 293 | 4 | 2.47 (0.36) |
| 322 | 4 | 2.27 (0.32) |
| 4045 | 4 | 2.05 (0.07) |
| 8148 | 4 | 2.2 (0.23) |
| 834 | 4 | 2.83 (0.52) |
| 2303 | 3 | 2.57 (0.7) |
| 2320 | 3 | 2.41 (0.1) |
| 2977 | 3 | 2.71 (0.22) |
| 3006 | 3 | 2.31 (0.15) |
| 323 | 3 | 2.5 (0.54) |
| 5302 | 3 | 2.39 (0.4) |
| 578 | 3 | 2.59 (0.59) |
| 606 | 3 | 2.24 (0.38) |
| 6612 | 3 | 2.49 (0.24) |
| 6815 | 3 | 2.5 (0.2) |
| 860 | 3 | 2.07 (0.19) |
| 132 | 2 | 2.56 (0.46) |
| 149 | 2 | 2.59 (0.14) |
| 150 | 2 | 2.19 (0.17) |
| 156 | 2 | 2.11 (0.01) |
| 200 | 2 | 2.67 (0.28) |
| 227 | 2 | 2.73 (0.35) |
| 266 | 2 | 3.93 (1.74) |
| 276 | 2 | 2.33 (0.04) |
| 280 | 2 | 2.27 (0.37) |
| 3007 | 2 | 2.2 (0.18) |
| 306 | 2 | 2.11 (0.1) |
| 3421 | 2 | 2.49 (0.54) |
| 3783 | 2 | 2.04 (0.01) |
| 48 | 2 | 2.19 (0.05) |
| 525 | 2 | 1.98 (0.08) |
| 557 | 2 | 2.37 (0.01) |
| 7565 | 2 | 2.74 (0.21) |
| 815 | 2 | 2.22 (0.09) |
| 158 | 1 | 2.31 (-) |
| 236 | 1 | 2.81 (-) |
| 253 | 1 | 2.12 (-) |
| 256 | 1 | 2.67 (-) |
| 2761 | 1 | 1.99 (-) |
| 278 | 1 | 2.24 (-) |
| 3054 | 1 | 2.97 (-) |
| 3209 | 1 | 2.09 (-) |
| 379 | 1 | 1.99 (-) |
| 390 | 1 | 2.07 (-) |
| 441 | 1 | 1.97 (-) |
| 4590 | 1 | 2.93 (-) |
| 47 | 1 | 2.41 (-) |
| 4862 | 1 | 1.98 (-) |
| 527 | 1 | 2.3 (-) |
| 540 | 1 | 1.94 (-) |
| 65 | 1 | 2.2 (-) |
| 964 | 1 | 2.04 (-) |
