# Supplementary material for: Rapid Detection of Heterogeneous Vancomycin-Intermediate Staphylococcus aureus Based on Matrix-Assisted Laser Desorption Ionization Time-of-Flight: Using a Machine Learning Approach and Unbiased Validation
Source: Front Microbiol. 2018 Oct 11;9:2393. doi: 10.3389/fmicb.2018.02393 (PMC6193097; doi:10.3389/fmicb.2018.02393)
Supplement: Supplementary file 3 [file Table_3.DOCX]

**Supplementary Table 3**. The validation results of all the classifiers using nested 5-fold cross-validation, repeated 6 times

| **Folds** | **Repeat times** | **Area under the ROC curve** | | | |
| --- | --- | --- | --- | --- | --- |
|  |  | **K-nearest neighbor** | **Random forest** | **Decision tree** | **SVM with RBF kernel** |
| 1 | 1 | 0.690 | 0.679 | 0.841 | 0.762 |
| 2 | 1 | 0.671 | 0.821 | 0.790 | 0.873 |
| 3 | 1 | 0.667 | 0.722 | 0.591 | 0.778 |
| 4 | 1 | 0.813 | 0.889 | 0.742 | 0.897 |
| 5 | 1 | 0.766 | 0.746 | 0.560 | 0.730 |
| 1 | 2 | 0.738 | 0.770 | 0.484 | 0.778 |
| 2 | 2 | 0.857 | 0.857 | 0.647 | 0.889 |
| 3 | 2 | 0.663 | 0.508 | 0.726 | 0.659 |
| 4 | 2 | 0.671 | 0.754 | 0.599 | 0.746 |
| 5 | 2 | 0.742 | 0.778 | 0.524 | 0.770 |
| 1 | 3 | 0.631 | 0.631 | 0.603 | 0.627 |
| 2 | 3 | 0.833 | 0.857 | 0.615 | 0.873 |
| 3 | 3 | 0.667 | 0.754 | 0.762 | 0.778 |
| 4 | 3 | 0.746 | 0.698 | 0.619 | 0.873 |
| 5 | 3 | 0.786 | 0.976 | 0.702 | 0.865 |
| 1 | 4 | 0.813 | 0.802 | 0.639 | 0.841 |
| 2 | 4 | 0.758 | 0.849 | 0.659 | 0.849 |
| 3 | 4 | 0.536 | 0.675 | 0.754 | 0.683 |
| 4 | 4 | 0.643 | 0.714 | 0.615 | 0.746 |
| 5 | 4 | 0.865 | 0.778 | 0.722 | 0.960 |
| 1 | 5 | 0.627 | 0.770 | 0.667 | 0.770 |
| 2 | 5 | 0.611 | 0.639 | 0.560 | 0.690 |
| 3 | 5 | 0.643 | 0.750 | 0.548 | 0.738 |
| 4 | 5 | 0.746 | 0.730 | 0.675 | 0.730 |
| 5 | 5 | 0.841 | 0.921 | 0.893 | 0.817 |
| 1 | 6 | 0.694 | 0.722 | 0.663 | 0.794 |
| 2 | 6 | 0.635 | 0.643 | 0.746 | 0.683 |
| 3 | 6 | 0.802 | 0.794 | 0.738 | 0.778 |
| 4 | 6 | 0.722 | 0.857 | 0.710 | 0.889 |
| 5 | 6 | 0.786 | 0.821 | 0.635 | 0.833 |

SVM: support vector machine; RBF kernel: radial basis function kernel
