# Supplementary material for: Rapid Detection of Heterogeneous Vancomycin-Intermediate Staphylococcus aureus Based on Matrix-Assisted Laser Desorption Ionization Time-of-Flight: Using a Machine Learning Approach and Unbiased Validation
Source: Front Microbiol. 2018 Oct 11;9:2393. doi: 10.3389/fmicb.2018.02393 (PMC6193097; doi:10.3389/fmicb.2018.02393)

**Supplementary Figure 1. Bacterial composition of the MRSA isolates.**

SCC*mec* typing and MLST methods were used to discover the composition of VSSA and hVISA/VISA isolates. The result revealed a relatively diverse distribution in VSSA, whereas hVISA isolates showed a relative cluster regarding SCC*mec* and MLST typing. MLST: multilocus sequence typing; VSSA: vancomycin-susceptible *Staphylococcus aureus*; hVISA: heterogeneous vancomycin-intermediate *Staphylococcus aureus*; VISA: vancomycin-intermediate *Staphylococcus aureus*.


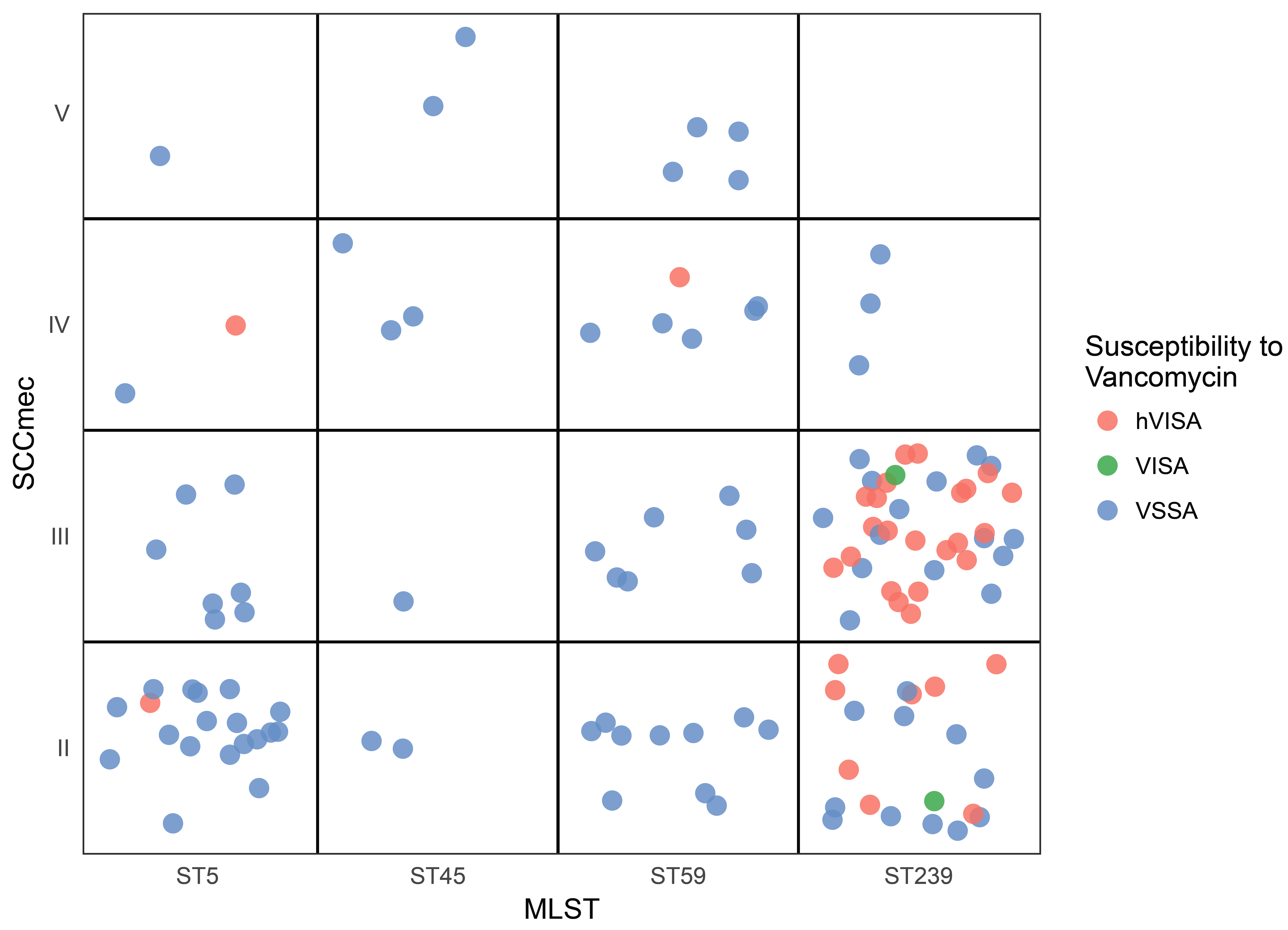

Supplement: Supplementary file 4 [file Data_Sheet_1.docx]
